# Distribution of Gb_3_ Immunoreactivity in the Mouse Central Nervous System

**DOI:** 10.3390/toxins2081997

**Published:** 2010-08-04

**Authors:** Fumiko Obata, Tom Obrig

**Affiliations:** Department of Microbiology and Immunology, University of Maryland School of Medicine, 685 W. Baltimore St. HSFI suite 380, Baltimore, MD 21201, USA; Email: tobrig@som.umaryland.edu

**Keywords:** globotriaosylceramide (Gb_3_), neuron, circumventricular organs (CVO), ependymal cells

## Abstract

We have shown previously that neurons in the mouse spinal cord express Gb_3_. We show in this article that distribution of anti-Gb_3_-Ab reactivity occurs in many different types of neurons of different areas of the central nervous system (CNS). The immunoreactive neurons are in olfactory bulbs, cerebral cortex, hippocampus, striatum, amygdala, thalamus, hypothalamus, cerebellum, and medulla oblongata. In several different circumventricular organs where vessels do not have the blood-brain-barrier (BBB) structure, anti-Gb_3_-Ab is not positive for vessel structures, while neurons at these regions are positive. Also, within the ventricular area, ependymal cells in the third ventricle express Gb_3_, as revealed by anti-Gb_3_-Ab staining and intensity analysis.

## 1. Introduction

In Shiga-toxin producing *Escherichia coli* (STEC) infections, a broad spectrum of central nervous system (CNS) symptoms occurs (abbreviations used in this article are listed in Table 1). Those symptoms include cortical blindness, poor fine-motor coordination, seizures and coma [1,2,3,4,5,6,7,8,9,10,11,12,13]. Globotriaosylceramide (Gb_3_) is a known receptor of Shiga toxin (Stx), which is central to the intoxication and disease process [14]. It has been shown that a Gb_3_ knockout mouse is resistant to Stx [15]. To understand target components within the CNS, determining which cell types express Gb_3_ is essential. Previously, we reported that in the mouse CNS, Shiga toxin-2 acts on spinal cord neurons which express Gb_3_, and leads to hindlimb paralysis [16]. Other mouse CNS cell types expressing Gb_3_ have not been described in detail.

**Table 1:** Abbreviations used in this manuscript.

Abbreviation	Abbreviated Term
AP	Area postrema ^(e)^
ARH	Arcuate nucleus of hypothalamus ^(c)^
BBB	Blood-brain-barrier
BLA	Basolateral nucleus of the amygdala ^(c)^
CNS	Central nervous system
CP	Caudate-putamen ^(a)^
CSF	Cerebro-spinal fluid
CVO	Circumventricular organs
DMX	Dorsal motor nucleus of the vagus ^(e)^
ec	External capsule ^(c)^
MD	Mediodorsal nucleus of the thalamus ^(d)^
ME	Median eminence ^(c)^
MH	Medial habenula ^(d)^
NTS	Nucleus of the solitary tract ^(e)^
OVLT	Organum vasculosum of the lamina terminalis ^(b)^
PVT	Paraventricular nucleus of the thalamus ^(d)^
SCO	Subcommissural organ
SFO	Subfornical organ ^(d)^
V3	Third ventricle
V4	Fourth ventricle
VL	Lateral ventricle
XII	Hypoglossal nucleus ^(e)^

(a) See Figure 2d for the positional information; (b)See Figure 4b for the positional information; (c) See Figure 4d for the positional information, (d) See Figure 4f for the positional information.

The trafficking route of Stx into the CNS is as important as determining its target. In human STEC patients’ brain magnetic resonance imaging (MRI), regions as the basal ganglia and also thalamus, cerebellum and brain stem, are found positive for increased permeability of fluid [17,18,19,20]. In a rabbit model, MRI showed enhanced permeability in the area surrounding V3 after Stx injections [21]. However, precise Stx trafficking routes and the mechanisms involved are still in question. Circumventricluar organs (CVO) are known to be devoid of the blood-brain-barrier (BBB), thus exchange of substances between the plasma and the CNS parenchyma is relatively easy [22]. The CVO is situated around the V3 (OVLT, SFO, ME, posterior pituitary, pineal gland and SCO) as well as the V4 (AP). Also, the choroid plexus located at both V3 and V4, is sometimes considered as the CVO. If the vessels at the CVO are expressing Gb_3_, it may increase the chance of being the primary target in the CNS. In this article, Gb_3_ expression in the CVO is addressed. Ependymal cells form a lining of the ventricle, which separates cerebro-spinal fluid (CSF) and parenchyma. As the choroid plexus makes CSF from serum and secretes it into the ventricles, there is a possibility of Stx2 in serum being transferred to the ventricle. If ependymal cells express Gb_3_, this also could be an entry point of Stx into the CNS parenchyma. 

## 2. Materials and Methods

### 2.1. Animals

Specific pathogen-free C57BL/6 mice, male, 20–22 g body weight (b.w.) were purchased from Charles River (Wilmington, MA, USA). Mice were given food and water *ad libitum*. All procedures were approved by the University of Maryland School of Medicine Animal Care and Use Committee. A total of 5 mice were used in this study.

### 2.2. Tissue Harvesting

Mice were euthanized by CO_2_ inhalation. Two mice were perfused with 20 mL saline, followed by 20 mL 4% paraformaldehyde/phosphate buffered saline (4% PFA/PBS). Brains were marked for the Bregma position (the crossing point of the coronal suture and the sagittal suture on the skull) with a knife incision. Brains and spinal cords were harvested, and further fixed in 4% PFA/PBS overnight at room temperature. Brains and spinal cords from 3 mice were fixed in the same manner without perfusion. Brains were trimmed to 2 mm thickness from the Bregma to both rostral and caudal ends. Spinal cords were trimmed into cervical, thoracic and lumbar segments. After incubating in 30% sucrose/PBS at 4 °C overnight, trimmed segments were sectioned to 50 μm thickness using a sliding microtome (SM2000R, Leica Microsystems, Bannockburn, IL, USA). The positions of brain sections from the Bregma was determined with reference to a C57BL/6 brain atlas [23]. Sections were collected and held in PBS at 4 °C until use.

### 2.3. Immunofluorescence Staining of Free-Floating Sections

Staining was done according to Obata *et al.* [16] and Kolling *et al.* [24]. Antibodies used in this study were anti-Gb_3_ monoclonal antibody (MAb) (Beckman Coulter, Brea, CA, USA), anti-NeuN MAb (a neuronal marker, Millipore, Billerica, MA, USA) and Cy3 conjugated anti-GFAP MAb (an astrocytic marker, Sigma-Aldrich, St. Louis, MO, USA), at dilutions of 1:100, 1:1000, and 1:1000, respectively. For isotype matched controls, rat IgM (Millipore) and mouse IgG_1_ (Millipore) were used at dilutions of 1:100 and 1:100, respectively. 4’,6-Diamidino-2-phenylindole (DAPI) was used to visualize nuclei. A Zeiss LSM510 microscope (Carl Zeiss Inc., Thornwood, NY, USA) was utilized in this study.

### 2.4. Intensity Analysis of Anti-Gb_3_ Immunofluorescence

For line profile intensity analysis, a LSM510 software line profile function was used. Within one image, a fixed length line was used to collect intensity data. Three intensity samples of neuronal and ependymal cytoplasm were collected per image. Appearance of high intensity of 2000 or higher was expressed as percentage of total data collected from the line profile. The VL, V3 and central canal regions were analyzed. Six images from each area were taken randomly from 3 mice, which were selected from a total of 5 mice (either perfuse-fixed or none perfuse-fixed). Averages of high intensity (%) between neurons and ependymal cells were compared. For area intensity analysis, Image-Pro Plus software (MediaCybernetics, Inc., Silver Spring, MD, USA) was used. The image acquired by LSM510 was saved as Tagged Image File Format (TIFF) in RGB, and only green channel (Gb_3_-AlexaFluor488) was retained in the image using Adobe Photoshop 7.0 (Adobe Systems Inc., San Jose, CA, USA). The TIFF image was converted to Gray scale, and a fixed area (μm^2^) of region of interest (ROI) was made using Image-Pro Plus. The intensity range higher than background pixels was chosen, and 3 ROI areas within neuronal or ependymal cytoplasm per image were taken as intensity samples. The equivalent regions and number of samples comparable to those used in the Line profile intensity analysis were analyzed by the area intensity analysis method. Averages of ROI areas between neurons and ependymal cells were compared. 

### 2.5. Statistics

Data from line profile or area intensity analysis were analyzed by student t-test (paired two-tail test), and *p*-values less than 0.01 were determined as significant.

## 3. Results and Discussion

**Figure 1:**
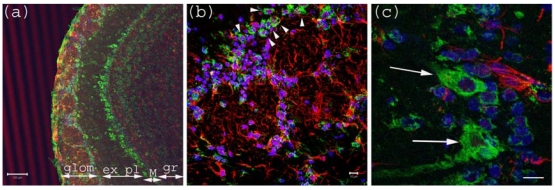
Anti-Gb_3_-Ab staining of Olfactory bulb. (**a**) Olfactory bulb sections stained with anti-Gb_3_-Ab (Green), and anti-GFAP-Ab (Red) and DAPI (Blue). Glomerular layer (glom), external plexiform (ex pl), Mitral cell layer (M) and granular layer (gr) are visible as neurons are stained anti-Gb_3_. Bar indicates 100 μm. (**b**) High magnification of glomerular layer. Arrowheads point to anti-Gb_3_-Ab reactive periglomerular neurons. Bar indicates 10 μm. (**c**) High magnification of Mitral cell layer. Arrows show anti-Gb_3_-Ab positive Mitral cells. Bar indicates 10 μm.

**Figure 2:**
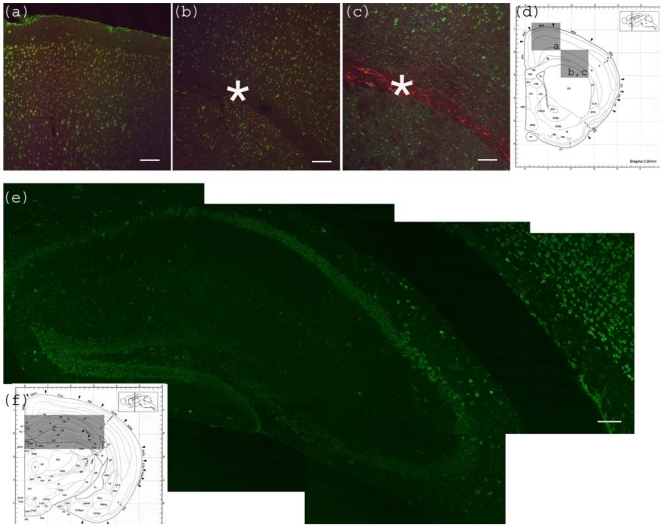
Anti-Gb_3_-Ab staining of cerebrum. (**a**) Motor cortex region showing anti-Gb_3_ stain (Green) in 2-6 layers of neurons, which are also positive for neuron specific anti-NeuN-Ab (Red). Position of the image corresponding to the Bregma 0.90 mm is shown in (d) as a square. (**b**) Corpus callosum region of the same section is shown. Astrocyte rich corpus callosum (*) is anti-Gb_3_ and anti-NeuN negative. Lowerleft area to corpus callosum is striatum (CP). Note that neurons of this area are also positive for anti-Gb_3_ Ab. Bar indicates 100 μm. (**c**) Corpus callosum region is stained with anti-Gb_3_-Ab (Green) and anti-GFAP-Ab (an astrocytic marker, Red). Contrast of GFAP (Red) positive layer versus cortex neurons (Green) is clear. Bar indicates 100 m. (**d**) Coronal architectural positions at Bregma 0.90 mm is shown with indication of images (a), (b) and (c) in squares. (**e**) Hippocampus region is shown with anti-Gb_3_-Ab (Green) stain. Neuronal layers of CA1, 2, 3 and dentate gyras are anti-Gb_3_ positive. Area in (e) is shown as a rectangle in (f). Bar indicates 100 m. (**f**) Coronal section architecture at Bregma −2.40 mm is shown. (d) and (f) are modified from [23].

**Figure 3:**
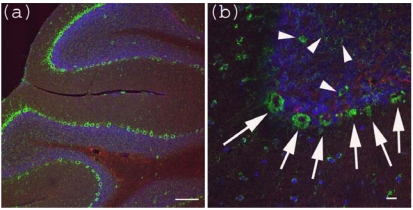
Anti-Gb_3_-Ab staining of cerebellum. (**a**) Cerebellum sections were stained with anti-Gb_3_ Ab (Green), anti-GFAP-Ab (Red) and DAPI (Blue). Perkinje layer is shown reactive to anti-Gb_3_-Ab. Bar indicates 100 μm. (**b**) Higher magnification of (a). Arrows indicate anti-Gb_3_-Ab positive Perkinje cells, while arrowheads indicate anti-Gb_3_-Ab positive neurons in the granule layer. Bar indicates 10 μm.

**Figure 4:**
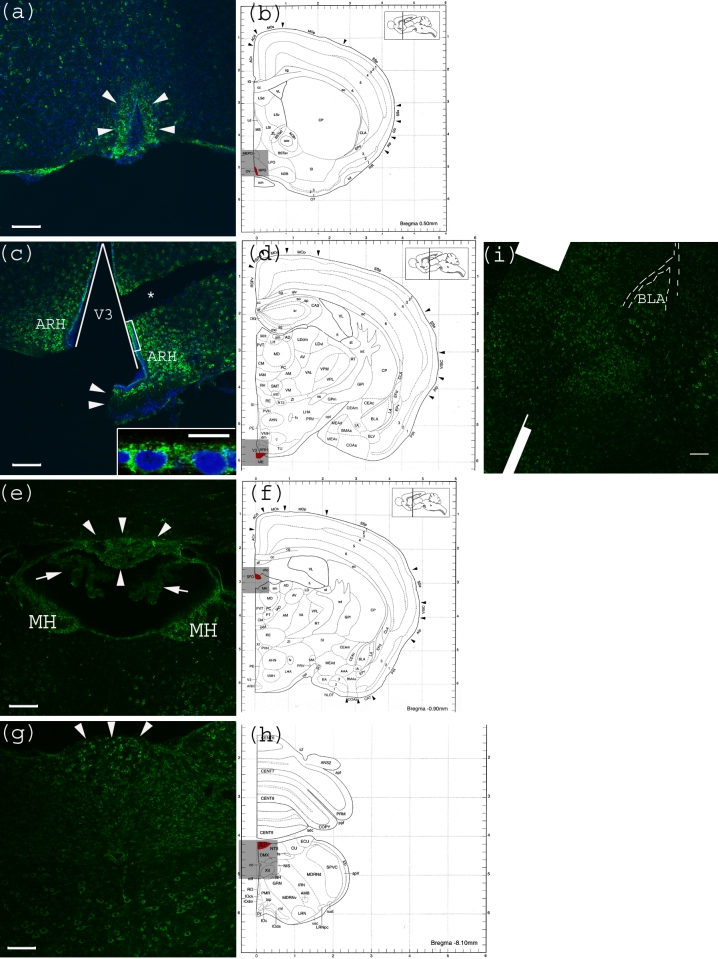
Anti-Gb_3_-Ab staining of CVO areas (**a**) Anti-Gb_3_-Ab stain (Green) of OVLT region, which is depicted in (b) as Red. Arrowheads indicate the OVLT area; (**b**) Architectural structure of mouse Bregma 0.50 mm; (**c**) Anti-Gb_3_-Ab stain (Green) of a region containing ME, which is depicted in (d) as Red. Arrowheads indicate the ME area. Neurons in ARH, which are adjacent to ME are anti-Gb_3_-Ab reactive. The V3 is shown as a triangular area. A part of ependymal layer (square region) is shown in inset with higher magnification. (*) indicates a broken tissue space; (**d**) Architectural structure of mouse Bregma −1.30 mm; (**e**) Anti-Gb_3_-Ab stain (Green) of a region containing SFO, which is depicted in (f) as Red. Arrowheads indicate the SFO area, while arrows show anti-Gb_3_-Ab negative choroid plexus. Neurons of MH are also anti-Gb_3_-Ab reactive. Lower area of this field includes thalamic nuclei as PVT and MD, in which neurons are positive for anti-Gb_3_-Ab. (**f**) Architectural structure of mouse Bregma -0.90 mm; (**g**) Anti-Gb_3_-Ab stain (Green) of a region containing AP, which is depicted in (h) as Red. Arrowheads indicate AP area. Neurons of several different nuclei in the medulla oblongata such as NTS, DMX and XII are also anti-Gb_3_-Ab positive; (**h**) Architectural structure of mouse Bregma −8.10 mm; (**i**) Neurons in the amygdala are also anti-Gb_3_-Ab positive. BLA is indicated in the picture. Dotted line outlines external capsule (ec). See architectural structure in (d). All Bars indicate 100 m, except an inset in (c) which indicates 10 m. Blue color in (a) and (c) indicates nuclei staining using DAPI. (b), (d), (f) and (h) are modified from [23]. See abbreviations in Table 1.

Anti-Gb_3_-Ab reactive neurons were seen throughout the mouse CNS. In the olfactory bulb, where Mitral cells accept input from nasal epithelium cells to sense smell, and interneurons such as periglomerular cells and granule cells reside, all neurons were Gb_3_ positive (Figure 1). In the cerebrum, neurons in the cortex, including motor cortex were also Gb_3_ positive (Figure 2a). Astrocytes in the corpus callosum which is close to the cerebral cortex were Gb_3_ negative (Figure 2b,c). Hippocampus neurons, CA1, 2, 3 and dentate gyrus, were Gb_3_ positive (Figure 2e). Also, the neurons in the striatum (Figure 2b), amygdala (Figure 4i), thalamus (Figure 4e) and hypothalamus were Gb_3_ positive. The hypothalamic nuclei (ARH) neurons are shown to be Gb_3_ positive in Figure 4c. In the cerebellum, Perkinje cells as well as granule cells were also Gb_3_ positive (Figure 3). Neurons throughout the mouse CNS appeared to express Gb_3_, hence, depending on the CNS entry site of Stx, all these neurons could become targets of the toxin. 

CVO areas in the mouse CNS were also tested for anti-Gb_3_-Ab reactivity. Within the tested CVO areas, none appeared to have a Gb_3_ expressing vessel structure. In contrast, neurons of these areas were positive for Gb_3_. In the OVLT, neurons which have characteristic large nuclei, were shown to be Gb_3_ positive (Figure 4a). In Figure 4c, the lining of V3 consisting of ependymal cells appears positive for Gb_3_. In Figure 4e, neurons in the SFO, as well as MH are Gb_3_ positive, while the choroid plexus is Gb_3_ negative. In Figure 4g, neurons in the medulla oblongata are Gb_3_ positive including the AP area. Within this image, a central canal positioned in the center, appears very dim. 

**Figure 5:**
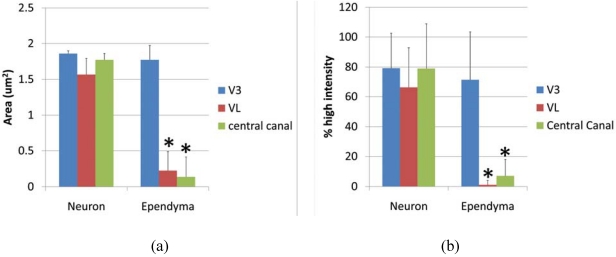
Intesnsity of anti-Gb_3_-Ab staining in neurons *vs.* ependyma. (**a**) High intensity area comparison of neurons and ependymal cells from V3, VL or central canal. Values from each region (neuron *vs.* ependyma) are compared, and the statistically significant difference is shown as (*). (**b**) High intensity percentage from Line profile analysis comparing neurons and ependymal cells from V3, VL or central canal. Values from each region (neuron *vs.* ependyma) are compared, and statistically significant differences are shown as (*). * indicates *p* < 0.01 in 2-tailed, paired t-test.

Ependymal cells were tested as a Gb_3_ expressing cell type and intensity analysis was performed comparing high intensity pixels in neuron and ependyma in the ventricle area as V3, VL and central canal. A square shaped region of interest (ROI) was used to detect the pixel area which is higher than background intensity. The average area (μm^2^) of neurons *vs.* ependymal cells in V3, VL and central canal was; 1.857 ± 0.039 *vs.* 1.773 ± 0.197 (p = 0.275), 1.567 ± 0.085 *vs.* 0.226 ± 0.262 (p = 0.0097), 1.7739 ± 0.085 *vs.* 0.137 ± 0.275 (p = 0.0014), respectively. *P* values less than 0.01 were considered to be statistically significant, and shown as (*) in Figure 5a. The line profile intensity analysis revealed a similar result that averages of high intensity percent of neurons *vs.* ependyma in V3, VL and central canal were; 79.23 ± 23.29 *vs.* 71.41 ± 32.03 (p = 0.289), 66.31 ± 26.46 *vs.* 1.156 ± 2.997 (p = 4.029E-10), 77.73 ± 30.39 *vs.* 4.346 ± 4.954 (p = 1.042E-08). The results are graphed in Figure 5b. In both analyses, the intensity level of Gb_3_ positive neurons and ependymal cells in the V3 region was similar, thus ependymal cells in this area were Gb_3_ positive. In contrast, VL and central canal ependymal cells were Gb_3_ negative. 

As the CVO is naturally leaky, serum Stx2 might travel through this route where vessels do not express Gb_3_. As ependymal cells at V3 express Gb_3_, this could serve as an entry point of Stx, if Stx enters into CSF in the mouse CNS. Trafficking of Stx in the CNS needs to be further investigated.

## 4. Conclusions

Mouse CNS neurons from various areas exhibited anti-Gb_3_-Ab reactivity. Ependymal cells at V3 were also found to be positive for anti-Gb_3_-Ab reactivity. However, vessels at the CVO or other areas of the mouse CNS did not exhibit anti-Gb_3_ reactivity.
